# Biliary atresia-specific deciduous pulp stem cells feature biliary deficiency

**DOI:** 10.1186/s13287-021-02652-8

**Published:** 2021-11-22

**Authors:** Soichiro Sonoda, Koichiro Yoshimaru, Haruyoshi Yamaza, Ratih Yuniartha, Toshiharu Matsuura, Erika Yamauchi-Tomoda, Sara Murata, Kento Nishida, Yoshinao Oda, Shouichi Ohga, Tasturo Tajiri, Tomoaki Taguchi, Takayoshi Yamaza

**Affiliations:** 1 Department of Molecular Cell Biology and Oral Anatomy, Kyushu University Graduate School of Dental Science, 3-1-1 Maidashi, Higashi-ku, Fukuoka, 812-8582 Japan; 2 Department of Pediatric Surgery, Kyushu University Graduate School of Medical Sciences, Fukuoka, Japan; 3 Department of Pediatric Dentistry, Kyushu University Graduate School of Dental Science, Fukuoka, Japan; 4 Department of Anatomy, Faculty of Medicine, Public Health, and Nursing, Universitas Gadjah Mada, Jogjakarta, Indonesia; 5 Department of Oral and Maxillofacial Radiology, Kyushu University Graduate School of Dental Science, Fukuoka, Japan; 6 Department of Anatomic Pathology, Kyushu University Graduate School of Medical Sciences, Fukuoka, Japan; 7 Department of Pediatrics, Kyushu University Graduate School of Medical Sciences, Fukuoka, Japan; 8 Fukuoka College of Health Sciences, Fukuoka, Japan

**Keywords:** Biliary atresia, Patient-derived human deciduous pulp stem cells, Bile duct regeneration

## Abstract

**Background:**

Biliary atresia (BA) is a severe hepatobiliary disease in infants that ultimately results in hepatic failure; however, its pathological mechanism is poorly elucidated. Current surgical options, including Kasai hepatoportoenterostomy and orthotopic liver organ transplantations, are palliative; thus, innovation in BA therapy is urgent.

**Methods:**

To examine whether BA-specific post-natal stem cells are feasible for autologous cell source for BA treatment, we isolated from human exfoliated deciduous teeth, namely BA-SHED, using a standard colony-forming unit fibroblast (CFU-F) method and compared characteristics as mesenchymal stem cells (MSCs) to healthy donor-derived control SHED, Cont-SHED. BA-SHED and Cont-SHED were intrasplenically transplanted into chronic carbon tetrachloride (CCl_4_)-induced liver fibrosis model mice, followed by the analysis of bile drainage function and donor integration in vivo. Immunohistochemical assay was examined for the regeneration of intrahepatic bile ducts in the recipient’s liver using anti-human specific keratin 19 (KRT19) antibody.

**Results:**

BA-SHED formed CFU-F, expressed MSC surface markers, and exhibited in vitro mesenchymal multipotency similar to Cont-SHED. BA-SHED showed less in vitro hepatogenic potency than Cont-SHED. Cont-SHED represented in vivo bile drainage function and KRT19-positive biliary regeneration in chronic carbon tetrachloride-induced liver fibrosis model mice. BA-SHED failed to show in vivo biliary potency and bile drainage function compared to Cont-SHED.

**Conclusion:**

These findings indicate that BA-SHED are not feasible source for BA treatment, because BA-SHED may epigenetically modify the underlying prenatal and perinatal BA environments. In conclusion, these findings suggest that BA-SHED-based studies may provide a platform for understanding the underlying molecular mechanisms of BA development and innovative novel modalities in BA research and treatment.

**Supplementary Information:**

The online version contains supplementary material available at 10.1186/s13287-021-02652-8.

## Background

Biliary atresia (BA) is a severe hepatobiliary disease in infants with persistent jaundice, alcoholic stools, dark urine, and high levels of serum bilirubin [1]. If left untreated, BA leads to fibrosis, cirrhosis, and ultimately hepatic failure, resulting in rapid deterioration of infant health, failure to thrive, and death by one or two years of life. BA is characterized by obstructive cholestasis associated with progressive fibroinflammatory obliteration of the extrahepatic biliary tree and rapid progression of intrahepatic biliary fibrosis [2]. BA is managed by surgical options, including Kasai hepatoportoenterostomy (KHPE) and orthotopic liver organ transplantations (OLT). KHPE is essential for long-term survival with the native liver to recover bile drainage and liver fibrosis [3]. OLT is the only option for patients who do not respond or have life-threatening complications with or without KHPE. Thus, innovation in BA therapy is urgent.

Human deciduous pulp stem cells were first identified in the dental pulp tissues of exfoliated deciduous teeth, namely stem cells from human exfoliated deciduous teeth (SHED) [4]. SHED express mesenchymal stem cell (MSC) characteristics, including cell proliferation, multipotency, and immunosuppressive function [5]. Considering the recent development of the in vitro hepatic potency and in vivo anti-fibroinflammatory and hepatic regenerative effects [6, 7], SHED may offer a novel modality for intractable liver diseases such as BA [8]. Stem cell transplantation has become a more feasible alternative to OLT [9]. Autologous transplantation may be more beneficial to BA than allogenic transplantation due to reduced surgical morbidity, limited immunosuppression-related toxicity, and increased cell engraftment [10].

BA causes deciduous tooth damage, including green pigmentation and dentin hypoplasia, and induces cell death and dentinogenic dysfunction in healthy donor-derived control SHED, referred to as Cont-SHED, via the AKT, extracellular regulated kinase 1/2, and nuclear factor kappa B pathways [11]. However, the stem cell properties and therapeutic potency of BA-patient-derived SHED, referred to as BA-SHED, are not well understood. Thus, this study aimed to demonstrate our hypothesis that BA-SHED is a feasible candidate as an autologous donor for BA research and therapy. We investigated the in vitro stem cell properties and hepatobiliary potency of BA-SHED. We also investigated the therapeutic efficacy of BA-SHED in chronically carbon tetrachloride (CCl_4_)-damaged fibrotic liver model mice.

## Methods

### Animals

Wild-type C57BL/6J mice (male, 8 weeks old) were obtained from the Charles River Laboratory Japan (Yokohama, Japan). The animals were housed individually and freely fed with sterile water and standard chow (MF diet, Oriental Yeast, Tokyo, Japan) under controlled environmental conditions with a 12 h light/12 h dark cycle.

### Isolation and culture of BA-SHED

The colony-forming unit fibroblast (CFU-F) method [12] was used to isolate BA-SHED and Cont-SHED. The dental pulp tissues of exfoliated human deciduous teeth from BA patients (*n* = 3, 5–7 years old) and healthy donors (*n* = 3, 5–7 years old) were digested with 0.3% collagenase type I (Worthington Biochemicals, Lakewood, NJ, USA) and 0.4% dispase II (FUJIFILM Wako Pure Chemicals, Osaka, Japan) for 60 min at 37 °C and passed through a 70-μm cell strainer to obtain single cells. Single cells were seeded in T-75 culture flasks and washed with sterilized Ca^2+^-free and Mg^2+^-free phosphate-buffered saline (PBS) 3 h after seeding. The adherent cells were incubated in a complete growth medium. The complete growth medium consisted of 15% fetal bovine serum (FBS; Equitech-Bio, Kerrville, TX, USA), 100 µM L-ascorbic acid 2-phosphate (FUJIFILM Wako Pure Chemicals), 2 mM L-glutamine (Nacalai Tesque, Kyoto, Japan), and premixed antibiotics containing 100 U/mL penicillin and 100 µg/mL streptomycin (premixed P/S; Nacalai Tesque) in minimum essential medium alpha (Thermo Fisher Scientific, Waltham, MA, USA) for 16 days. The CFU-F-formed cells were passaged at 1.5 × 10^5^ cells per dish on 100-mm culture dishes, and passage 3 (P3) BA-SHED and Cont-SHED were used for further experiments.

### Characterization of BA-SHED

BA-SHED and Cont-SHED (P3) were characterized by the criteria of MSCs, including plastic adherent colony formation, surface antigen expression, and multilineage adipogenic, chondrogenic, and odontogenic/osteogenic differentiation, as described previously [13]. Isolated mononuclear cells (1.0 × 10^4^ cells per flask) were seeded in T-75 flasks and cultured in a complete growth medium for 16 days. The colonies formed were stained with a solution of 4% paraformaldehyde and 0.1% toluidine blue in PBS (pH 7.4). The expressions of CD146, CD105, CD90, CD73, CD45, CD34, CD19, CD14, CD11b, and HLA-DR were examined in P3 BA-SHED by flow cytometric (FCM) analysis. The cells were maintained under adipogenic, chondrogenic, and odontogenic/osteogenic culture conditions. The expressions of adipocyte-, chondrocyte-, and odontoblast/osteoblast-specific genes (*peroxisome proliferator activated receptor gamma 2* [*PPARG2*] and *lipoprotein lipase* [*LPL*] for adipocytes, *SRY-box 9 transcription factor* [*SOX9*] and *collagen type X alpha 1* [*COL10A1*] for chondrocytes, and *runt-related transcription factor 2* [*RUNX2*] and *bone gamma-carboxyglutamate protein* [*BGLAP*] for odontoblasts/osteoblasts) were analyzed by quantitative reverse transcription polymerase chain reaction (RT-qPCR). Specific staining for adipogenesis, chondrogenesis, and odontogenesis/osteogenesis was analyzed by Oil Red O, Alcian blue, and Alizarin Red S staining, respectively. Cell proliferation was analyzed by cell viability, bromodeoxyuridine uptake, and population doubling assays. Telomerase activity was evaluated using a qPCR-based telomeric repeat amplification protocol using a TeloTAGGG Telomerase PCR ELISA kit (Merck, Darmstadt, Germany) according to the manufacturer’s instructions. Heat-inactivated samples were used as the negative controls.

### Induction of BA-SHED into hepatocyte-like cells, BA-SHED-Heps

BA-SHED and Cont-SHED (P3; 2.5 × 10^5^ cells per dish) were seeded on human fibronectin-coated 100-mm culture dishes (Corning, Corning, NY, USA) and maintained in complete growth medium until they reached confluence. The cultured cells were treated in a sequential hepatogenic medium based on Iscove’s modified Dulbecco’s medium (IMDM; Thermo Fisher Scientific) containing premixed P/S (Nacalai Tesque). The BA-SHED cells were preincubated with human epidermal growth factor (20 ng/mL; PeproTech, Rocky Hill, NJ, USA) and human fibroblast growth factor 2 (FGF2; 10 ng/mL; PeproTech) for 2 days. They were then stimulated with FGF2 (10 ng/mL; PeproTech), hepatocyte growth factor (HGF; 20 ng/mL; PeproTech), and nicotinamide (5 mmol/L; Merck) for 7 days and then cultured with oncostatin M (20 ng/mL; PeproTech), dexamethasone (1 µmol/L; Merck), and ITS + premix (50 mg/mL; Thermo Fisher Scientific) for 21 days as previously described [7]. The medium was changed twice a week. The expressions of hepatocyte-specific genes and hepatic function were assessed.

### Hepatocyte-specific gene expression analysis in BA-SHED-Heps

Total RNA was extracted from cultured BA-SHED-Heps and Cont-SHED-Heps. Target genes were analyzed by RT-qPCR. Cont-SHED and BA-SHED were used as controls.

### Immunofluorescence of BA-SHED-Heps

Cultured cells were blocked with 5% normal donkey serum (Thermo Fisher Scientific) and incubated with the primary antibodies, followed by treatment with Alexa Fluor 488- or Alexa Fluor 568-conjugated secondary antibodies (1:200; Thermo Fisher Scientific). The cells were then stained with 4', 6-diamidino-2-phenylindole (DAPI; 1 µg/ml; Thermo Fisher Scientific) and observed under an AxioVert light microscope (Carl Zeiss Microscopy, Jena, Germany). The primary antibodies used are summarized in Additional file 1: Table S1.

### Secretion and hepatic metabolic assays of BA-SHED-Heps

BA-SHED-Heps and Cont-SHED-Heps were washed with Hanks’ balanced salt solution (HBSS) without phenol red (Nacalai Tesque) and incubated for 48 h in a freshly prepared hepatogenic culture medium. The hepatogenic culture medium contained FGF2 (10 ng/mL; PeproTech), HGF (20 ng/mL; PeproTech), and nicotinamide (0.61 g/L; Merck) in IMDM (Thermo Fisher Scientific). Some cultures were incubated with indirect bilirubin (25 nM; Merck) in HBSS for 60 min. The conditioned medium (CM) was collected from cultures. The contents of ALB, glucose, triglycerides, urea, and bilirubin in the CMs were measured by ELISA and colorimetric analysis.

### Hepatic kinetic assay in BA-SHED-Heps

BA-SHED-Heps and Cont-SHED-Heps were incubated with indocyanine green (ICG; 1 mg/mL; Merck) in IMDM (Thermo Fisher Scientific) for 1 h. After washing with HBSS (Nacalai Tesque), the cells were cultured for 6 h. The cells were incubated overnight with 0.1% bovine serum albumin (BSA; Merck) in IMDM (Thermo Fisher Scientific) and treated for 5 h with Dil-conjugated acetylated low-density lipoprotein (Dil-Ac-LDL; 10 μg/mL; Cell Applications, San Diego, CA, USA) and 0.1% BSA (Merck) in IMDM (Thermo Fisher Scientific). BA-SHED-Heps and Cont-SHED-Heps were also incubated with cholyl-lysyl-fluorescein (CLF; 5 μM; BD Bioscience, Franklin Lake, NJ, USA) in HBSS (Nacalai Tesque) at 37 °C for 15 min. All samples were fixed with 4% paraformaldehyde and stained with DAPI (1 µg/ml; Thermo Fisher Scientific). All samples were imaged with an Axio Imager M.2 fluorescent microscope equipped with an Apotome 2 (Carl Zeiss Microscopy). Cont-SHED and BA-SHED were used as controls.

### CYP3A4 activity test in BA-SHED-Heps

BA-SHED-Heps and Cont-SHED-Heps were incubated with or without dexamethasone (50 µM; Merck) for 24 h. CYP3A4 activity in BA-SHED-Heps and Cont-SHED-Heps was analyzed by a spectrometric assay using a P450-Glo CYP3A4 Kit with Luciferin-IPA (Promega, Madison, WI, USA). Results were measured with a GloMax Navigator luminometer (Promega) and shown as normalized relative light unit values to the untreated well in each culture condition according to the manufacturer’s instructions.

### Transplantation of BA-SHED into CCl_4_-treated chronic liver fibrosis model mice

CCl_4_ was freshly mixed with olive oil (CCl_4_:olive oil = 1:4 volume/volume; FUJIFILM Wako Pure Chemicals) immediately before administration. The CCl_4_ solution (1.0 mL/kg body weight) or olive oil (1.0 mL/kg body weight; FUJIFILM Wako Pure Chemicals) was intraperitoneally injected into mice twice a week for eight weeks. BA-SHED and Cont-SHED (1 × 10^6^ in 100 µL PBS per mouse) or PBS (100 µL) were infused into 4-week-CCl_4_-treated mice via the spleen. All mice were maintained under immunosuppressant-free conditions throughout the experiment.

### In vivo assays for hepatic fibroinflammatory biomarkers in BA-SHED transplanted mice

Serum was harvested from mice 8 weeks after CCl_4_ administration. Serum AST and ALT levels were measured by colorimetric assay using a Transaminase CII-Test Kit (FUJIFILM Wako Pure Chemicals) according to the manufacturer’s instructions. The expressions of *alpha-smooth muscle actin 2, smooth muscle, aorta* (*Acta2*), *type I collagen alpha 1 chain* (*Col1a1*), *matrix metalloprotease* (*Mmp9*), *Mmp2*, *tissue inhibitor of metalloproteinase 1* (*Timp1*), *Timp2,* interleukin 6 (*Il6*), *transforming growth factor-beta* (*Tgfb*), and *tumor necrosis factor-alpha* (*Tnfa*) were examined in mouse liver tissues by RT-qPCR. Serum human albumin (ALB) and bilirubin levels were measured by enzyme linked immunosorbent assay (ELISA) and colorimetric assay.

### In vivo* monitoring of transplanted donor cells in CCl4 treated mice*

BA-SHED and Cont-SHED labeled with XenoLight DiR NIR fluorescent dye (DiR; 10 μg/mL; Perkin Elmer, Waltham, MA; 1 × 10^6^ in 100 µL PBS) or PBS (100 µL PBS) were intrasplenically infused into 4-week-CCl_4_ treated mice. Ventral images of the mice were obtained 24 h after infusion with an optical in vivo imaging system IVIS Lumina III (Perkin Elmer) using living image software (Perkin Elmer).

### Histological and immunohistochemical analyses in BA-SHED transplanted mice

Mouse livers were harvested 8 weeks after CCl_4_ administration. The livers were fixed with 4% paraformaldehyde in PBS and processed for paraffin sectioning. The paraffin sections were stained with hematoxylin and eosin and Sirius Red. Paraffin sections were incubated with 3% hydrogen peroxide in methanol for 30 min and treated with 5% normal goat serum (Thermo Fisher Scientific) in PBS. They were incubated with primary antibodies (Additional file 1: Table S1) overnight at 4 °C, followed by treatment with Dako Envision + system-HRP labeled polymer anti-rabbit or anti-mouse (Agilent, Santa Clara, CA). The sections were then visualized with 0.05% diaminobenzidine-4HCl (Dojindo Laboratories, Kumamoto, Japan) and 0.006% hydrogen peroxide and counter-stained with hematoxylin. Immunohistochemical controls were performed with non-immune mouse IgG_1_, mouse IgG_2a_, and rabbit IgG, instead of primary antibodies.

### Double immunofluorescent analysis in BA-SHED transplanted mice

Sections were treated with primary antibodies using an Opal 3-plex kit (Perkin Elmer, Waltham, MA, US) according to the manufacturer’s instructions and stained with DAPI (1 μg/ml; Thermo Fisher Scientific). All samples were imaged with an Axio Imager M.2 fluorescent microscope equipped with an Apotome 2 (Carl Zeiss Microscopy).

### FCM analysis

Cultured cells (0.1 × 10^6^ cells per 100 µL HBSS) were suspended in ice-cold FCM buffer. The FCM buffer consisted of 2% heat-inactivated FBS (Merck) in HBSS (Nacalai Tesque). The cell suspension was incubated with R-phycoerythrin (R-PE)-conjugated primary antibodies (1 µg per 100 µL HBSS; Additional file 1: Table S2) at 4 °C for 45 min and measured on a FACSVerse flow cytometer (BD Biosciences, Franklin Lake, NJ, USA). As controls, isotype-matched antibodies conjugated with R-PE were used instead of primary antibodies. The percentage of positive cells was determined using FACSuite software (BD Biosciences) compared to control cells stained with corresponding isotype-matched antibodies in which a false-positive rate of less than 1% was accepted.

### RT-qPCR assay

RNA samples were extracted from the cell and tissue samples using TRIzol reagent (Thermo Fisher Scientific), digested with DNase I (Promega), and purified using an RNeasy Mini Kit (Qiagen). cDNA was prepared from the purified total RNA by reverse transcription using a Revertra Ace qPCR kit (TOYOBO, Tokyo, Japan) according to the manufacturer’s instructions and used for RT-qPCR assays. Gene expression was analyzed by RT-qPCR using the cDNA mixed with EagleTaq Master Mix (Roche Applied Science, Babaria, Germany) and target TaqMan probes (Thermo Fisher Scientific; Additional file 1: Tables S3 and S4) with a Light Cycler 96 system real-time PCR cycler (Roche Applied Science). The PCR steps were as follows: preincubation 1 at 50 °C for 120 s, preincubation 2 at 95 °C for 600 s, two-step amplification at 95 °C for 15 s, and 60 °C for 60 s (45 cycles). Human and mouse 18S ribosomal RNA were used for normalization.

### ELISA and colorimetric assay

The total protein concentration of samples was quantified using a Bio-Rad protein assay (Bio-Rad Laboratories, Hercules, CA, USA) to ensure equal loading of each well. ELISA and colorimetric assay were performed using commercially available kits (Additional file 1: Table S5), according to the manufacturer’s instructions. The results were measured using a Multiskan GO microplate reader (Thermo Fisher Scientific).

### Statistical analysis

All tests were carried out in triplicates or more. Statistical results are expressed as the mean ± standard error of mean (SEM). Comparisons between two groups were performed using independent two-tailed Student’s *t*-tests. Multiple group comparisons were analyzed by one-way repeated measures analysis of variance followed by Tukey’s post hoc test. Differences were considered statistically significant at *P* < 0.05. All statistical analyses were performed using PRISM 6 software (GraphPad, Software, La Jolla, CA, USA).

## Results

### BA-SHED displays MSC characteristics in vitro

**Fig. 1:**
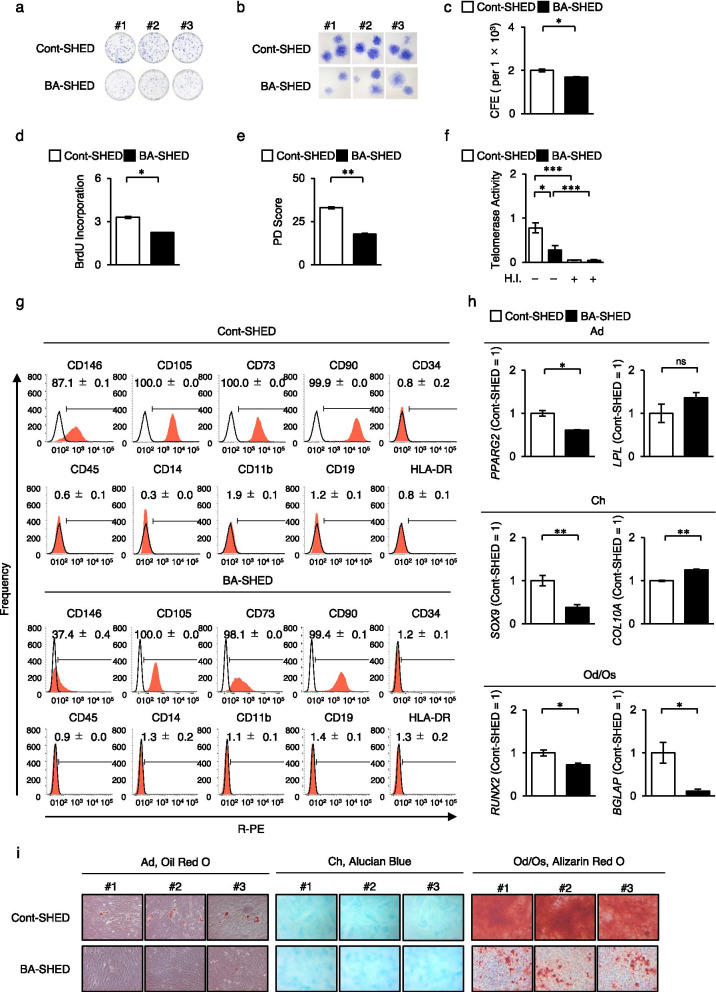
Characterization of stem cells from human exfoliated deciduous teeth from biliary atresia patients (BA-SHED) and healthy donors (Cont-SHED). **a**–**c** Representative images show the attached colonies by toluidine blue staining. Lower (**a**) and higher (**b**) magnification. The graph shows the colony-forming efficiency (CFE) per 1 × 10^5^ seeding cells (**c**). **d**, **e** The graphs show the cell proliferation ability by bromodeoxyuridine incorporation (**d**) and population doubling (PD; **e**) assays. (**f**) The graph shows the telomerase activity by telomerase activity test. H.I.: heat inactivation. (**g**) Representative histograms of cell surface antigens were analyzed by flow cytometric analysis. The numbers indicate the means ± standard error of mean (SEM) of a positive rate of target markers. Areas filled with red; target antibody-stained histograms; solid lines; isotype-matched control-stained histograms. **h**, **i** Multipotency after adipogenic (Ad), chondrogenic (Ch), and odontogenic/osteogenic (Od/Os) induction. The graphs show the expression of lineage-specific genes by reverse transcription quantitative polymerase chain reaction (RT-qPCR). The results are shown as ratios to Cont-SHED (Cont-SHED = 1). *BGLAP*, *bone gamma-carboxyglutamine protein*; *Col10a1*, *type X collagen, alpha 1*; *LPL*, *lipoprotein lipase*; *PPARG2*, *peroxisome proliferator activated receptor gamma 2*; *RUNX2*, *runt-related transcription factor 2*; *SOX9*, *SRY-box 9* (**h**). Representative images show the lineage-specific matrix formation by Oil Red O, Alcian blue, and Alizarin Red S staining (**i**). **c**–**f**, **h**: *n* = 3 for all groups. *P < 0.05, **P < 0.01, ***P < 0.005. The graph bars represent the means ± standard error of the mean (SEM)

BA-SHED were isolated from three patients with BA (5–7 years old) using the CFU-F method. BA-SHED formed plastic-adherent colonies but showed a reduced colony frequency compared to Cont-SHED (Fig. 1a–c). BA-SHED exhibited reduced proliferation potential compared to Cont-SHED by bromodeoxyuridine uptake and population doubling assays (Fig. 1d, e). Telomerase activity was lower in BA-SHED than in Cont-SHED (Fig. 1f). BA-SHED displayed a similar immunophenotype to Cont-SHED by FCM analysis (Fig. 1g, Additional file 1: Fig. S1). The primitive MSC marker CD146 was lower in BA-SHED than in Cont-SHED (Fig. 1g, Additional file 1: Fig. S1). BA-SHED and Cont-SHED expressed low levels of antigenic markers by FCM analysis and exhibited low immunogenicity by lymphocyte-mixed reaction test (Additional file 1: Fig. S2). BA-SHED displayed in vitro multipotency into adipocytes, chondrocytes, and odontoblasts/osteoblasts under lineage-specific induction conditions, as indicated by the expression of lineage-specific genes, including *PPARG2*, *LPL*, *SOX9*, *COL10A1*, *RUNX2*, and *BGLAP*, by RT-qPCR analysis and formation of lineage-specific matrix by Oil-Red O, Alcian blue, and Alizarin-Red S staining (Fig. 1h, i). The odontogenic/osteogenic capacity of BA-SHED was lower than that of Cont-SHED (Fig. 1h, i). These findings indicate that BA-SHED fulfilled the criteria for MSCs.

### BA-SHED exhibit a hepatogenic potency in vitro

**Fig. 2:**
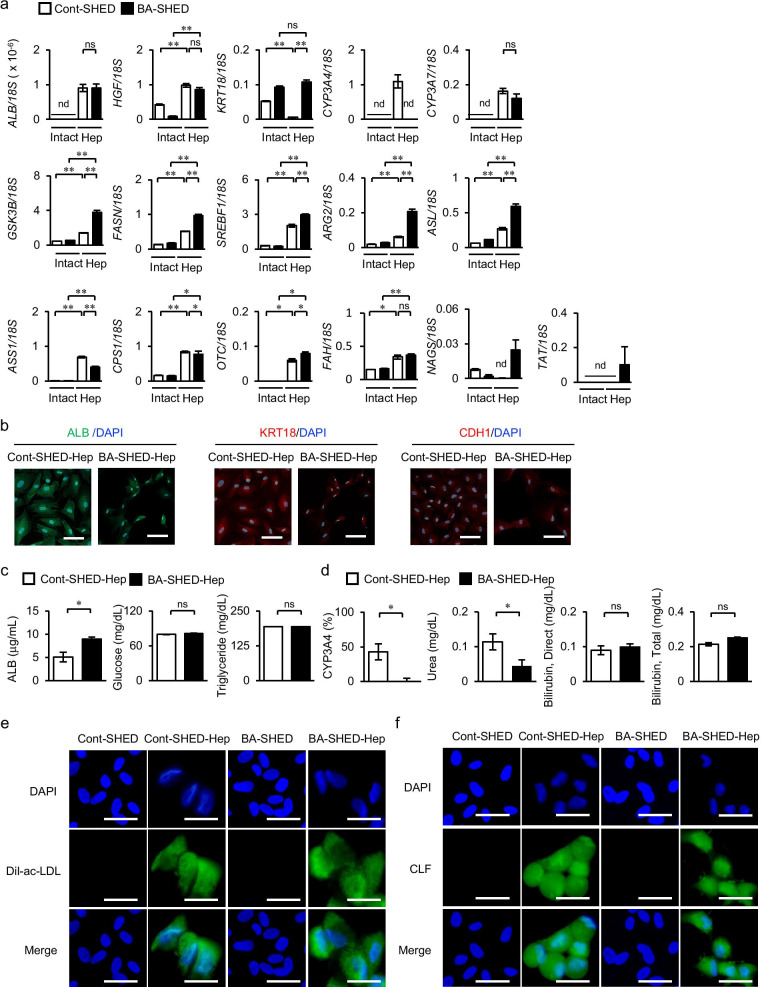
Hepatic properties and functions of hepatocyte-like cells converted from BA-SHED (BA-SHED-Heps) and Cont-SHED (Cont-SHED-Heps). **a** The graphs show the gene expression of hepatocyte-specific genes by RT-qPCR. *ALB*, *albumin*; *ARG2*, *arginase 2*; *ASL*, *argininosuccinate lyase*; *ASS1*, *argininosuccinate synthase 1*; *CPS1*, *carbamoyl-phosphate synthase 1*; *CYP3A7*, *cytochrome P450 3A4*; *CYP3A7*, *cytochrome P450 3A7*; *FAH*, *fumarylacetoacetate hydrolase*; *FASN*, *fatty acid synthase*; *GSK3B*, *glycogen synthase kinase 3 beta*; *HGF*, *hepatocyte growth factor*; *KRT18*, *keratin 18*; *NAGS*, *N-acetyl glutamate synthase*; *OTC*, *ornithine transcarbamylase*; *SREBF1*, *sterol regulatory element-binding transcriptional factor 1*; *TAT*, *tyrosine aminotransferase*. Results are shown as ratios to 18S ribosomal RNA (/*18S*). **b** Representative images show the expression of ALB, KRT18, and cadherin 1 (CDH1) by immunofluorescent analysis. **c** The graphs show the amount of ALB (left panel), glucose (middle panel), and triglyceride (right panel) in the conditioned medium (CM) of cultures by ELISA and colorimetry assay. **d** The graph shows the xenobiotic activity of CYP3A4 under dexamethasone loading and the amount of urea, total bilirubin, and direct bilirubin in the CM by colorimetry assay. The results of CYP3A4 activity are shown as ratios to untreated cultures. **e**, **f** Representative images show uptake of Dil-fluorescent dye-conjugated acetylated low-density lipoprotein (Dil-ac-LDL) and cholyl-lysyl-fluorescein (CLF). **a**, **c**, **d**: *n* = 3 for all groups. *P < 0.05, **P < 0.01, ***P < 0.005. nd, no detection; ns, no significance. Graph bars represent means ± SEM. **b**, **e**, **f**: Nuclei were stained with 4', 6-diamidino-2-phenylindole (DAPI). Scale bars, 30 µm

Considering the in vitro hepatogenic capacity of SHED into immature hepatocyte-like cells under hepatogenic induction conditions with the stimulation of hepatogenic cytokines [7], BA-SHED were induced into hepatocyte-like cells, referred to as BA-SHED-Heps, and the in vitro hepatic characteristics were analyzed. BA-SHED-Heps expressed higher levels of hepatocyte-specific genes, including *ALB*, *HGF*, *keratin 18* (*KRT18*), *cytochrome P450 3A7 (CYP3A7)*, *glycogen synthase kinase 3 beta* (*GSK3B*), *fatty acid synthase* (*FASN*), *sterol regulatory element-binding transcriptional factor 1* (*SREBF1*), *arginase 2* (*ARG2*), *argininosuccinate lyase* (*ASL*), *argininosuccinate synthase 1* (*ASS1*), *carbamoyl-phosphate synthase 1* (*CPS1*), *ornithine transcarbamylase* (*OTC*), *fumarylacetoacetate hydrolase* (*FAH*), *N-acetyl glutamate synthase* (*NAGS*), and *tyrosine aminotransferase* (*TAT*), in comparison with non-induced intact BA-SHED by RT-qPCR (Fig. 2a). BA-SHED-Heps exhibited higher expression levels of *KRT18*, *CYP3A7*, *GSK3B*, *FASN*, *SREBF1*, *ARG2*, *ASL*, *OTC*, *NAGS*, *and TAT*, but a similar expression of *HGF*, *ASS1*, and *CPS1*, compared to Cont-SHED-converted immature hepatocyte-like cells, Cont-SHED-Heps (Fig. 2a). BA-SHED did not express *CYP3A4* but exhibited a similar expression of *CYP3A7* to Cont-SHED-Heps (Fig. 2a). Immunohistochemical analysis detected ALB, KRT18, and cadherin 1 (CDH1) in both BA-SHED-Heps and Cont-SHED-Heps (Fig. 2b).

BA-SHED-Heps and Cont-SHED-Heps exhibited the endocytotic and exocytotic abilities of ICG (Additional file 1: Fig. S3). BA-SHED-Heps showed the secreting capacity of ALB, glucose, and triglycerides into the CM (Fig. 2c). BA-SHED-Heps showed no xenobiotic activity of CYP3A4, less production of urea, and similar secretion of direct and total bilirubin compared to Cont-SHED-Heps (Fig. 2d). BA-SHED-Heps, but not BA-SHED or Cont-SHED, showed intracellular accumulation of LDL and bile acid by Dil-Ac-LDL and CLF treatments (Fig. 2e, f). These findings indicated that BA-SHED exhibited less hepatogenic potency than Cont-SHED.

### BA-SHED transplantation ameliorates liver fibrosis in chronically CCl_4_-treated mice

**Fig. 3:**
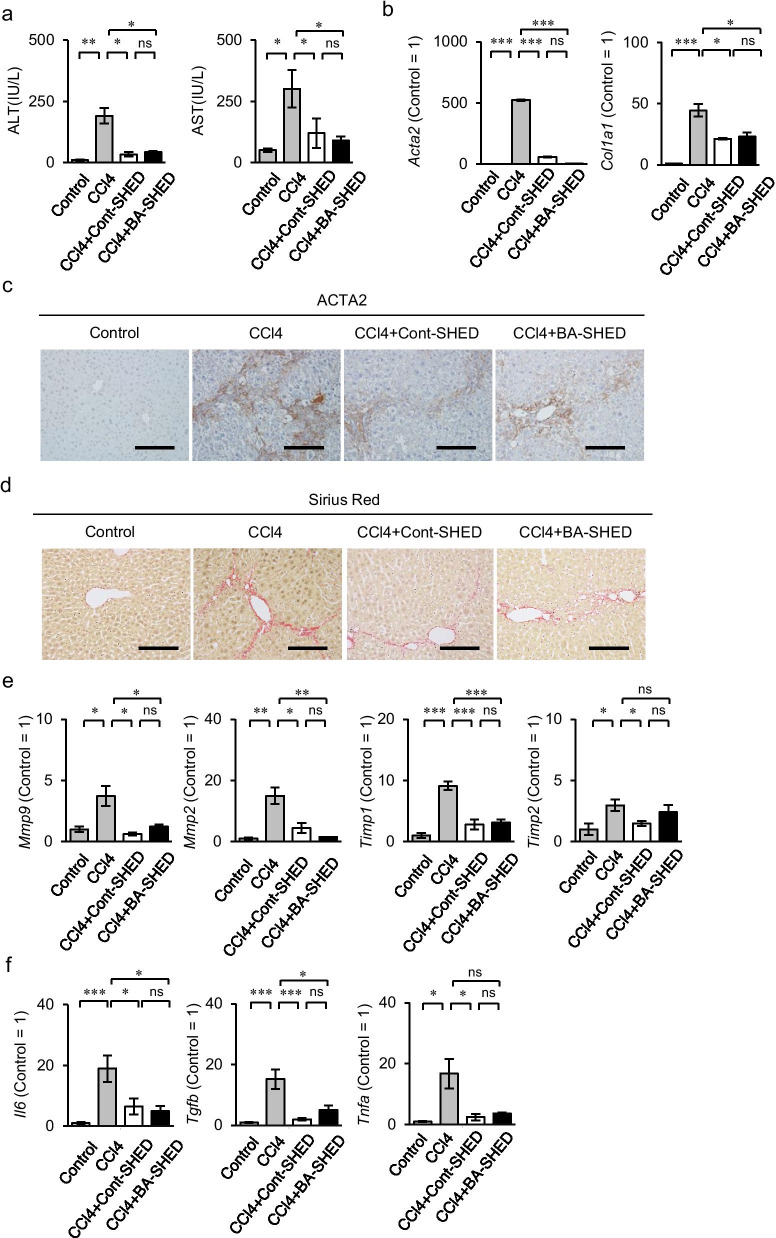
Effects of BA-SHED transplantation on liver fibrosis in CCl_4_-treated mice. Mice were harvested 8 weeks after CCl_4_ treatment. **a** The graphs show the serum levels of aspartate aminotransferase (AST) and alanine aminotransferase (ALT) by colorimetry. **b** The graphs show the expression of *alpha-smooth muscle actin 2, smooth muscle, aorta* (*Acta2*) and *type I collagen alpha 1 chain* (*Col1a1*) in livers by RT-qPCR. **c** Representative images of ACTA2 distribution in livers were detected by immunohistochemical analysis. Sections were counterstained with hematoxylin. **d** Representative images of the liver were analyzed by Sirius Red staining. **e**, **f** The graphs show the gene expression of fibrogenesis-related markers (**e**) and inflammation-related markers (**f**) in livers by RT-qPCR. E: *Mmp2*, *matrix metalloproteinase 2*; *Mmp9*, *matrix metalloproteinase 9*; *Timp1*, *tissue inhibitor of metalloproteinase 1*; *Timp2*, tissue inhibitor of metalloproteinase 1. F: *Il6*, *interleukin 6*; *Tgfb*, *transforming growth factor-beta*; *Tnfa*, *tumor necrosis factor-alpha*. **a**–**f**: Control, olive oil-treated mice; CCl4, CCl_4_ treated mice; CCl4 + Cont-SHED, Cont-SHED transplanted CCl_4_ treated mice; CCl4 + BA-SHED, BA-SHED transplanted CCl_4_ treated mice. **a**, **b**, **e**, **f**: *n* = 9 for all groups. *P < 0.05, **P < 0.01, ***P < 0.005. ns, no significance. The graph bars represent the means ± SEM. **b**, **e**, **f**: The results are shown as ratios to the control group (Control = 1). **c, d**: Scale bars, 200 µm

BA-SHED and Cont-SHED (1 × 10^6^ per mouse) were intrasplenically infused into 4-week-CCl_4_-treated mice, referred to as BA-SHED and Cont-SHED mice, respectively. All mice were analyzed 8 weeks after CCl_4_ treatment. Biochemical assays showed that the serum levels of aspartate aminotransferase (AST) and alanine aminotransferase (ALT) in the CCl_4_ mice were reduced in both the BA-SHED and Cont-SHED mice (Fig. 3a). The livers of BA-SHED and Cont-SHED mice, referred to as BA-SHED and Cont-SHED livers, respectively, showed decreased gene expression of *Acta2* and *Col1a1* compared to the livers of CCl_4_ mice, referred to as CCl_4_ livers, by RT-qPCR (Fig. 3b). Immunohistochemical analysis and Sirius Red staining revealed decreased ACTA2-positive cells and fibrous tissue deposition in the BA-SHED and Cont-SHED livers (Fig. 3c, d). The BA-SHED and Cont-SHED livers showed reduced levels of fibroinflammation-related marker genes, including *Mmp*, *Mmp2*, *Timp1*, *Timp2, Il6*, *Tgfb*, and *Tnfa*, compared to the CCl_4_ livers by RT-qPCR (Fig. 3e, f).

### Transplanted donor BA-SHED integrates into the liver tissue of chronically CCl_4_-treated mice

**Fig. 4:**
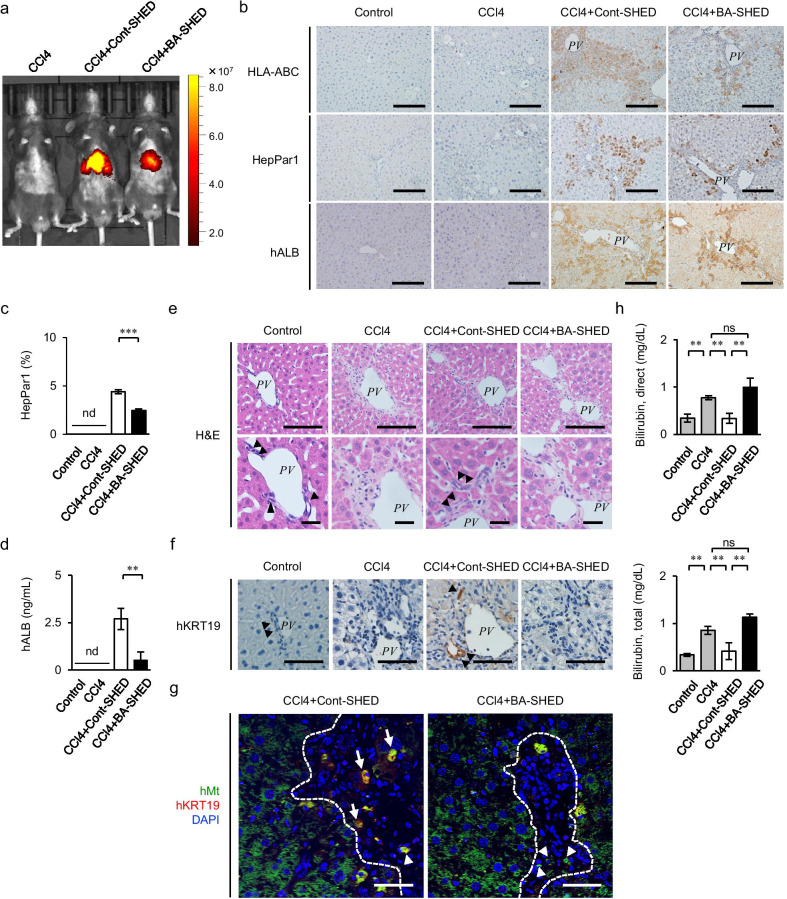
Transplanted donor BA-SHED regenerate the parenchyma, but not the intrahepatic bile ducts in the livers of recipient CCl_4_-treated mice. **a** Representative images of in vivo kinetics of Cont-SHED and BA-SHED were detected in CCl_4_-treated mice 24 h after transplantation by DiR labeling. **b–h** Mice were harvested 8 weeks after CCl_4_ treatment. Representative images of human leukocyte antigen A, B, and C (HLA-ABC), hepatocyte paraffin 1 antigen (HepPar1), and human ALB (hALB) in the liver were detected by immunohistochemical staining (**b**). The graph shows the HepPar1-positive area in mouse livers by immunohistochemical analysis (**c**). The graph shows the serum levels of hALB in mice by ELISA (**d**). Representative images of livers were analyzed by hematoxylin and eosin (H&E) staining (**e**). Representative images of human KRT19 (hKRT19) in livers were detected by immunohistochemical staining. Nuclei were stained by hematoxylin (**f**). Representative images of hKRT19 and human mitochondria (hMt) in livers were detected by double immunofluorescent staining. Nuclei were stained by DAPI (**g**).The graphs show the serum levels of direct and total bilirubin in mice by colorimetry (**h**). **a**–**g**: Control, olive oil-treated mice; CCl4, CCl_4_ treated mice; CCl4 + Cont-SHED, Cont-SHED transplanted CCl_4_ treated mice; CCl4 + BA-SHED, BA-SHED transplanted CCl_4_ treated mice. **b**, **e**–**g**: Scale bars, 200 µm. *PV*, portal vein. arrowheads, biliary duct. **c**, **d**, **h**: *n* = 9 for all groups. **P < 0.01, ***P < 0.005. nd, no detection; ns, no significance. The graph bars represent the means ± SEM

Using in vivo imaging, the fluorescence intensity of DiR-labeled donor cells was detected in the right upper quadrant of the abdomen corresponding to the BA-SHED and Cont-SHED livers, but not in the CCl_4_ mice, 1 day after transplantation (Fig. 4a). Immunohistochemical analysis using human-specific antibodies showed that human leukocyte antigens A, B, and C, human hepatocyte-specific paraffin 1 (HepPar1), and human ALB-positive cells were detected in the parenchymal periphery of the BA-SHED and Cont-SHED livers, but not in the non-treated and CCl_4_ livers (Fig. 4b, c). The HepPar1 positive area was lower in the BA-SHED livers than in the Cont-SHED livers (Fig. 4c). Serum human ALB levels were lower in the BA-SHED mice than in the Cont-SHED mice, but not in the non-treated and CCl_4_ mice, as determined by ELISA (Fig. 4d).

### BA-SHED transplantation does not rescue biliary excretion in chronically CCl_4_-treated mice

Histological analysis showed that the intrahepatic bile ducts in the periportal region of mouse livers disappeared in the CCl_4_ livers (Fig. 4e). The reconstruction of intrahepatic bile ducts was observed in the periportal region of the Cont-SHED livers, but not in that of the BA-SHED livers (Fig. 4e). Immunohistochemical analysis using a specific antibody against human KRT19 (hKRT19) revealed hKRT19-positive cells in the periportal region of the Cont-SHED livers, but not in those of the BA-SHED, CCl_4_, and control livers (Fig. 4f). Double immunofluorescent analysis showed that hKRT19-positive cells were coexpressed with hMt in the periportal region of the Cont-SHED livers, but not in those of the BA-SHED (Fig. 4g). Single hKRT19-positive cells were also found in the same region of the BA-SHED and Cont-SHED livers (Fig. 4g). Some single hMt-positive cells were also found in the parenchymal periphery of the BA-SHED and Cont-SHED livers (Fig. 4g). Cont-SHED transplantation reduced the increased serum levels of both total and direct bilirubin in CCl_4_-treated mice, but not BA-SHED transplantation (Fig. 4h). To understand the treatment discrepancy between Cont-SHED and BA-SHED, we speculated that the discrepancy may be caused by cellular epigenetic status. We then analyzed cellular levels of 5-mC DNA in BA-SHED and Cont-SHED by ELISA. BA-SHED exhibited the reduced level of 5-mC DNA compared to Cont-SHED (Additional file 1: Fig. S4), suggesting that biliary deficiency in BA-SHED transplantation may link the demethylated status of BA-SHED.

## Discussion

In this study we demonstrated in situ direct transdifferentiation capacity of healthy donor-derived SHED into cholangiocytes, as well as hepatocytes, in liver tissues under chronic liver fibrosis condition in CCl_4_-treated mice. To our knowledge, this is the first study demonstrating that biliary potency of healthy donor-derived SHED improves bile drainage and hepatobiliary function in chronically CCl_4_-damaged fibrotic liver model mice, suggesting that healthy donor-derived SHED are a feasible resource for therapeutic venues of BA treatment. Thus, these data further support that allografting healthy donor-derived SHED may be employed as “off the shelf” cell therapy for other hepatobiliary disorders.

This study demonstrated for the first time that SHED exhibited in vivo bipotency in hKRT19-positive cholangiocytes and HepPar1-positive hepatocytes in CCl_4_ damaged liver fibrous tissue. Moreover, the data suggest that SHED-mediated biliary regeneration plays a critical role in the treatment of liver fibrosis. However, the mechanism of SHED-mediated de novo biliary formation remains unclear. Delta-like ligand 4 induces the fate-deciding signal, NOTCH pathway, for cholangiocytes in hepatocyte progenitor cells (HPCs) [14] and accelerates the human bone marrow MSC-mediated biliary regeneration in a fulminant hepatic failure model study [15]. Meanwhile, donor SHED-Heps can directly reconstruct biliary ducts in CCl_4_ damaged liver tissue due to their transdifferentiation ability under TNFA stimulation [16]. Moreover, donor SHED may act as a niche to support the proliferation and cholangiocyte differentiation of HPCs via JAGGED-1-NOTCH2 signaling and the secretion of FGF7, as is the case with portal mesenchymal cells [17–19]. Thus, these findings suggest that SHED may orchestrally contribute to de novo bile duct formation in chronically injured livers. Further studies are necessary to clarify the cellular and molecular mechanisms of SHED-mediated cholangiogenesis.

The present study demonstrates the first generation of patient-derived BA-SHED. BA-SHED exhibited similar in vivo anti-fibroinflammatory effects and less in situ hepatic regeneration in chronically CCl_4_-treated mice compared to Cont-SHED; moreover, BA-SHED failed to induce in vivo biliary regeneration. Autologous induced pluripotent stem cell (iPSC)-derived neural cells clearly show the therapeutic advantages to both cell survival and immune response rather than allogenic iPSC-derived neural cells in the primate brains [20, 21]. Although the potential low immunogenicity of allogenic SHED reduces the immune response [13], allogenic rejection in recipients has never been eliminated. Autologous SHED transplantation is a novel regenerative approach for treating pulp diseases [22]. These findings suggest that BA-SHED are a feasible autologous cell source for BA treatment when biliary deficiency is rescued.

Diverse factors, such as genetic alternation, viral infections, and immune dysregulation, are implicated in the pathogenesis of BA at the prenatal and perinatal stages [23, 24]. However, the etiology of BA has not yet been fully elucidated. Given that the present BA-SHED transplant study demonstrated the deficiency of in vivo bile duct regeneration and the less reduction of hyperbilirubinemia in chronically CCl_4_-treated mice, BA-SHED are not feasible in the present animal models but are supposed to be a practical source for BA research and treatment. Basic helix-loop-helix family member A15 (BHLHA15) is involved in the commitment of HPCs into hepatocytes with high gene expression and metabolic activity of CYP3A4 accompanied by the suppression of cholangiogenic differentiation [25]. *Bhlha15* knockout mice promote pancreatitis by epigenetic reprogramming of genes via histone modification [26]. The gene expression and activity of CYP3A4 were silenced in BA-SHED-Heps. BA-SHED-Heps failed to express similar profile to CONT-SHED-Heps. These findings indicate that BA-SHED may exhibit hepatobiliary deficiency through epigenetic modification under prenatal and perinatal BA environments. The present in vitro odontogenic/osteogenic deficiency in BA-SHED may support the epigenetic regulation of BA-SHED under the BA environments.

The abnormal development of BA has been explained by developmental errors in the differentiation and morphogenesis of the bile duct system. Epigenetic modifications modify cell type-specific gene expression and regulate the development of various tissues and organs in the body [27]. Previous studies have shown that DNA hypomethylation alters interferon-gamma and CD11a expression in CD4-positive T cells of BA infants [28, 29] and causes biliary defects and interferon-gamma overexpression in zebrafish [30], suggesting that abnormal DNA methylation participates in the development of BA disorders. BA-specific iPSCs from peripheral blood cells show suppressed in vitro biliary potency but exhibit normal potency in HPCs [31, 32]. The present study evaluated that BA-SHED exhibited more demethylated DNA status than Cont-SHED. Thus, these findings suggest that the modified DNA methylation patterns of imprinted genes could change the present in vivo potency of biliary-specific deficiency and hepatic sufficiency in BA-SHED. However, genome-wide DNA methylation patterns and targeted DNA methylation in cholangiocyte-specific genes have not been analyzed in BA-SHED. Future DNA methylation analyses in BA-SHED could reveal the disease mechanism underlying BA and provide epigenetically engineered BA-SHED as a feasible autologous modality for BA treatment.

In conclusion, the present study demonstrated that SHED induced in vivo biliary duct and hepatocyte regeneration in chronically CCl_4_-induced liver fibrosis mice. On the contrary, BA-SHED showed biliary deficiency and reduced hepatic regeneration in mice with liver fibrosis, indicating that BA-SHED are not feasible source for BA treatment. The therapeutic discrepancy may be due to the epigenetic modification in BA-SHED underlying the prenatal and perinatal BA environment. Thus, these findings suggest that BA-SHED-based studies may provide an innovative platform for establishing disease models, revealing disease mechanisms, and developing novel modalities, such as disease treatment and drug screening, in BA. This disease-specific SHED-based approach may be a feasible tool for the innovation of disease mechanisms and effective therapies for various disorders.

## Supplementary Information

**Additional file 1**. Supplementay tables and figures.

## Data Availability

All data generated and analyzed during this study are included in this published article and its supplementary information files.

## Abbreviations

Acta2: Alpha-smooth muscle actin 2, smooth muscle, aorta
ALB: Albumin
ALT: Alanine aminotransferase
ARG2: Arginase 2
ASL: Argininosuccinate lyase
ASS1: Argininosuccinate synthase 1
AST: Aspartate aminotransferase
BA: Biliary atresia
BA-SHED: BA patient derived stem cells from human exfoliated deciduous teeth
BGLAP: Bone gamma-carboxyglutamate protein
BHLHA15: Basic helix-loop-helix family member A15
BSA: Bovine serum albumin
CCl_4_: Carbon tetrachloride
CDH1: Cadherin 1
CLF: Cholyl-lysyl-fluorescein
CFU-F: Colony-forming unit fibroblast
CM: Conditioned medium
COL1A1: Type I collagen alpha 1 chain
COL10A1: Collagen type X alpha 1
Cont-SHED: Healthy donor derived control stem cells from human exfoliated deciduous teeth
CPS1: Carbamoyl-phosphate synthase 1
CYP3A7: Cytochrome P450 3A7
DAPI: 4', 6-Diamidino-2-phenylindole
Dil-Ac-LDL: Dil-conjugated acetylated low-density lipoprotein
DiR: XenoLight DiR NIR fluorescent dye
ELISA: Enzyme linked immunosorbent assay
FAH: Fumarylacetoacetate hydrolase
FASN: Fatty acid synthase
FBS: Fetal bovine serum
FCM: Flow cytometry
FGF2: Fibroblast growth factor 2
FGF7: Fibroblast growth factor 7
5-mC: 5-Methylcytosine
GSK3B: Glycogen synthase kinase 3 beta
HBSS: Hanks’ balanced salt solution
HepPar1: Human hepatocyte-specific paraffin 1
HGF: Hepatocyte growth factor
hMt: Human mitochondria
HPCs: Hepatocyte progenitor cells
ICG: Indocyanine green
IMDM: Iscove’s modified Dulbecco’s medium
IL6: Interleukin 6
iPSC: Induced pluripotent stem cell
KHPE: Kasai hepatoportoenterostomy
KRT18: Keratin 18
KRT19: Keratin 19
LPL: Lipoprotein lipase
MMP9: Matrix metalloprotease 9
MMP2: Matrix metalloprotease 2
MSC: Mesenchymal stem cell
NAGS: N-acetyl glutamate synthase
OLT: Orthotopic liver organ transplantations
P3: Passage 3
PBS: Phosphate-buffered saline
premixed P/S: Premixed antibiotics containing 100 U/mL penicillin and 100 µg/mL streptomycin
SEM: Standard error of mean
SHED: Stem cells from human exfoliated deciduous teeth
SOX9: SRY-box 9 transcription factor
SREBF1: Sterol regulatory element-binding transcriptional factor 1
OTC: Ornithine transcarbamylase
PPARG2: Peroxisome proliferator activated receptor gamma 2
RT-qPCR: Quantitative reverse transcription polymerase chain reaction
RUNX2: Runt-related transcription factor 2
TAT: Tyrosine aminotransferase
TGFB: Transforming growth factor-beta
TIMP1: Tissue inhibitor of metalloproteinase 1
TIMP2: Tissue inhibitor of metalloproteinase 2.
TNFA: Tumor necrosis factor-alpha
