# Supplementary material for: Biliary atresia-specific deciduous pulp stem cells feature biliary deficiency
Source: Stem Cell Res Ther. 2021 Nov 22;12:582. doi: 10.1186/s13287-021-02652-8 (PMC8607730; doi:10.1186/s13287-021-02652-8)
Supplement: Supplementary file 1 — Additional file 1. Supplementay tables and figures. [file 13287_2021_2652_MOESM1_ESM.pdf]

Supplementary Information

Biliary atresia-specific deciduous pulp stem cells feature biliary deficiency

Soichiro Sonoda, Koichiro Yoshimaru, Haruyoshi Yamaza, Ratih Yuniartha, Toshiharu Matsuura, Erika Yamauchi-Tomoda, Sara Murata, Kento Nishida, Yoshinao Oda, Shouichi Ohga, Tatsuro Tajiri, Tomoaki Taguchi, Takayoshi Yamaza

Supplementary Methods

Immunogenicity assay of BA-SHED

The expression of immunogenic markers including cofactors (CD40, CD80, and CD86), T cells (CD3, CD4, CD8a, and CD28) were analyzed in P3 Cont-SHED and BA-SHED by flow cytometric analysis. Cell suspension was incubated with R-phycoerythrin (R-PE)-conjugated primary antibodies (1 μg per 100 μL Hanks' balanced salt solution (Nacalai Tesque, Kyoto, Japan); **Additional file 1: Supplementary Table 2**) at 4°C for 45 min and measured on a FACSVerse flow cytometer (BD Biosciences, Franklin Lake, NJ, USA). As controls, isotype-matched antibodies conjugated with R-PE were used instead of primary antibodies. The percentage of positive cells was determined using FACSuite software (BD Biosciences) compared to control cells stained with corresponding isotype-matched antibodies in which a false-positive rate of less than 1% was accepted.

For mixed lymphocyte reaction assay, Cont-SHED and BA-SHED (1.0×10^5 per well) and human PBMCs (1.0×10^5 per well) were preconditioned with gamma irradiation (30 Gy) under an MBR-1520R-3 X-ray irradiator (Hitachi, Tokyo, Japan).

Allogenic PBMCs (1.0 × 10^6 per well) were co-cultured with or without the preconditioned SHED or PBMNCs in a lymphocyte complete medium with or without phytohemagglutinin (PHA; 5 μg/mL, Merck, Darmstadt, Germany). The lymphocyte complete medium was consisted of 10% heat-inactivated FBS (Equitech-Bio, Kerrville, TX, USA), 2 mM L-glutamine (Nacalai Tesque), 1 mM sodium pyruvate (Nacalai Tesque), and premixed P/S (Nacalai Tesque) in RPMI-1640 medium (Thermo Fisher Scientific, Waltham, MA, USA) for 5 days. The cell viability of the PBMCs was assayed using a Cell Counting Kit-8 (Dojindo, Kumamoto, Japan) according to the manufacturer's instructions and measured using a Multiscan GO microplate spectrophotometer (Thermo Fisher Scientific).

Global DNA methylation assay

Genome DNA were collected form cultured cells and used for quantifying 5-methylcytosine (5-mC) levels using a commercially available kit (**Additional file 1: Supplementary Table 5**), according to the manufacturer's instructions. The results were measured using a Multiskan GO microplate reader (Thermo Fisher Scientific).

Supplementary Materials

Supplementary Table 1. The list of specific antibodies for immunohistochemistry and immunofluorescence.

Antibody names, antigens	Antibody types, hosts, clone names	Supplier names
anti-ACTA2 antibody, mouse	purified IgG2a, mouse, ASM-1/1A4	Merck
anti-ALB antibody, human	purified IgG, rabbit	Cell Signaling Technology
Anti-CDH1 antibody, human	purified IgG, rabbit	Cell Signaling Technology
anti-HepPar1 antibody, human	purified IgG1, mouse, OCH1E5	Abcam
anti-HLA-ABC antibody, human	purified IgG2a, mouse, W6/32	Abcam
anti-hMt antibody, human	purified, mouse IgG1, 113-1	Abcam
anti-KRT18 antibody, human	purified IgG1, mouse, CK-18	Abcam
anti-KRT19 antibody, human	purified IgG1, mouse, RCK108	Abcam
control mouse IgG1 kappa	purified IgG1 kappa, mouse, MOPC-21	Abcam
control mouse IgG2a kappa	purified IgG2a kappa, mouse, MOPC-173	Abcam
control rabbit IgG	purified IgG, rabbit	Abcam

ACTA2, actin, alpha 2, smooth muscle; ALB, albumin; CHD1, cadherin 1; HepPar1, human hepatocyte paraffin 1; HLA-ABC, human leukocyte antigens A, B, and C; hMt, human mitochondria; KRT18, cytokeratin 18; KRT19, cytokeratin 19.

Supplementary Table 2. The list of specific antibodies for flow cytometry

Antibody names, antigens	Antibody types, hosts, clone names	Supplier names
anti-CD3 antibody, human	R-PE-conjugated IgG2b kappa, mouse, HIT3a	BioLegend
anti-CD4 antibody, human	R-PE-conjugated IgG2b kappa, mouse, OKT4	BioLegend
anti-CD8a antibody, human	R-PE -conjugated IgG1 kappa, mouse, HIT8a	BioLegend
anti-CD11b antibody, human	R-PE -conjugated IgG1 kappa, mouse, HI111	BioLegend
anti-CD14 antibody, human	R-PE -conjugated IgG1 kappa, mouse, 63D3	BioLegend
anti-CD19 antibody, human	R-PE -conjugated IgG1 kappa, mouse, 47G	BioLegend
anti-CD28 antibody, human	R-PE -conjugated IgG1 kappa, mouse, CD28.2	BioLegend
anti-CD34 antibody, human	R-PE-conjugated IgG2a kappa, mouse, 561	BioLegend
anti-CD40 antibody, human	R-PE-conjugated IgG1 kappa, mouse, 5C3	BioLegend
anti-CD45 antibody, human	R-PE-conjugated IgG1 kappa, mouse, 2D1	BioLegend
anti-CD80 antibody, human	R-PE-conjugated IgG1 kappa, mouse, 2D10	BioLegend
anti-CD86 antibody, human	R-PE-conjugated IgG1 kappa, mouse, BU63	BioLegend
anti-CD90 antibody, human	R-PE-conjugated IgG1 kappa, mouse, 5E10	BioLegend
anti-CD73 antibody, human	R-PE-conjugated IgG1 kappa, mouse, AD2	BioLegend
anti-CD105 antibody, human	R-PE-conjugated IgG1 kappa, mouse, 43A3	BioLegend
anti-CD146 antibody, human	R-PE-conjugated IgG1 kappa, mouse, P1H112	BioLegend
anti-HLA-DR antibody, human	R-PE-conjugated IgG2a kappa, mouse, L243	BioLegend
control mouse IgG1 kappa	R-PE-conjugated IgG1 kappa, mouse, MOPC-21	BioLegend
control mouse IgG2a kappa	R-PE-conjugated IgG2a kappa, mouse, MOPC-173	BioLegend
control mouse IgG2b kappa	R-PE-conjugated IgG2a kappa, mouse, MPC-11	BioLegend

HLA-DR: human leukocyte antigen DR; R-PE: R-phycoerythrin

Supplementary Table 3. The list of TaqMan probes for human genes.

Gene names	Gene assay ID Numbers	Gene names	Gene assay ID Numbers
ALB	Hs00910225_m1	*HGF*	Hs00300159_m1
ARG2	Hs00982833_m1	*KRT18*	Hs02827483_g1
ASL	Hs00902699_m1	*LPL*	Hs00173425_m1
ASS1	Hs01597989_g1	*NAGS*	Hs00400246_m1
BGLAP	Hs01587814_g1	*OTC*	Hs00166892_m1
COL10A1	S00166657_m1	*PPARG*	Hs0115513_m1
CPS1	Hs00157048_m1	*RUNX2*	Hs00231692_m1
CYP3A4	Hs00604506_m1	*SOX9*	Hs01001343_g1
CYP3A7	Hs00426361_m1	*SREBF1*	Hs01088691_m1
FAH	Hs00164611_m1	*TAT*	Hs00356930_m1
FASN	Hs01005622_m1	18S rRNA	Hs99999901_s1
GSK3B	Hs01047719_m1		

ALB, albumin; ARG2, arginase 2; ASL, argininosuccinate lyase; ASS1, argininosuccinate synthase 1; BGLAP, bone gamma-carboxyglutamate protein; COL10A1, collagen, type X, alpha 1; CPS1, carbamoyl-phosphate synthase 1; CYP3A4: cytochrome P450 3 subfamily A member 4; CYP3A7: cytochrome P450 3 subfamily A member 7; FAH, fumarylacetoacetate hydrolase gene; FASN, fatty acid synthase; GSK3B, glycogen synthase kinase 3 beta gene; HGF, hepatocyte growth factor; KRT18, keratin 18; LPL, lipoprotein lipase; NAGS, N-acetylglutamate synthase; OTC, ornithine transcarbamylase; PPARG, peroxisome proliferator-activated receptor gamma; RUNX2, runt related transcription factor 2; SOX9, SRY-box9; SREBF1, sterol regulatory element binding transcriptional factor 1; TAT, tyrosine aminotransferase.

Supplementary Table 4. The list of TaqMan probes for mouse genes.

Gene names	Gene assay ID Numbers
Acta2	Mm00725412_s1
Col1a1	Mm00801666_g1
IL6	Mm00446190_m1
Mmp2	Mm00439498_m1
Mmp9	Mm00442991_m1
Tgfb1	Mm01178820_m1
Timp1	Mm01341361_m1
Timp2	Mm00441825_m1
Tnfa	Mm00443258_m1
18S rRNA	Mm03928990_g1

Actaa2, actin, alpha 2, smooth muscle; Col1a1, collagen, type I, alpha 1; Mmp2, matrix metalloproteinase 2; Mmp9, matrix metalloproteinase 9; Tgfb, transforming growth factor beta; Timp1, tissue inhibitor of metalloproteinase 1; Timp2, tissue inhibitor of metalloproteinase 2; Tnfa, tumor necrosis factor alpha.

Supplementary Table 5. The list of commercially available kits for colormetric and enzyme labelled immunosorbent assays.

Test names	Kit names	Supplier names
ALB	AssayMAX Human Albumin ELISA Kit	AssayPro
ALT, AST	Transaminase CII-Test Kit	FUJIFILM Wako Pure Chemicals
Bilirubin, direct and total	QuantiChrom Bilirubin Assay Kit	BioAssay Systems
5-mC DNA	MethylFlash Global DNA Methylation (5-mC/5-hmC) ELISA Easy Kit	Epigentek
Glucose	Glucose CII-test	FUJIFILM Wako Pure Chemicals
Triglyceride	Triglyceride E-test 1	FUJIFILM Wako Pure Chemicals
Urea	Urea Assay Kit	Abcam

ALB, albumin; ALT, alanine aminotransferase; AST, aspartate aminotransferase; CYP3A4: cytochrome P450 3 subfamily A member 4; 5-mC, 5- methylcytosine

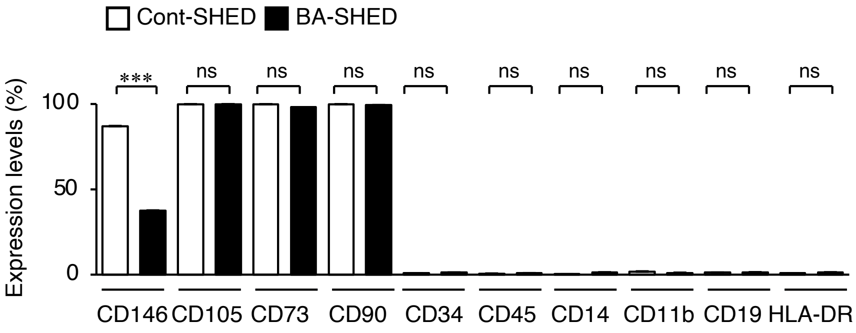

Supplementary Figure 1. Expression of mesenchymal stem cell (MSC) markers in stem cells from human exfoliated deciduous teeth from biliary atresia patients (BA-SHED) and healthy donors (Cont-SHED). The graphs show the expression levels (%) of MSC markers in Cont-SHED and BA-SHED by flow cytometric (FCM) analysis. n = 3 for all groups. HLA-DR, human leukocyte antigen DR. ***$P < 0.005$. ns, no significance. The graph bars represent the means ± standard error of mean (SEM).

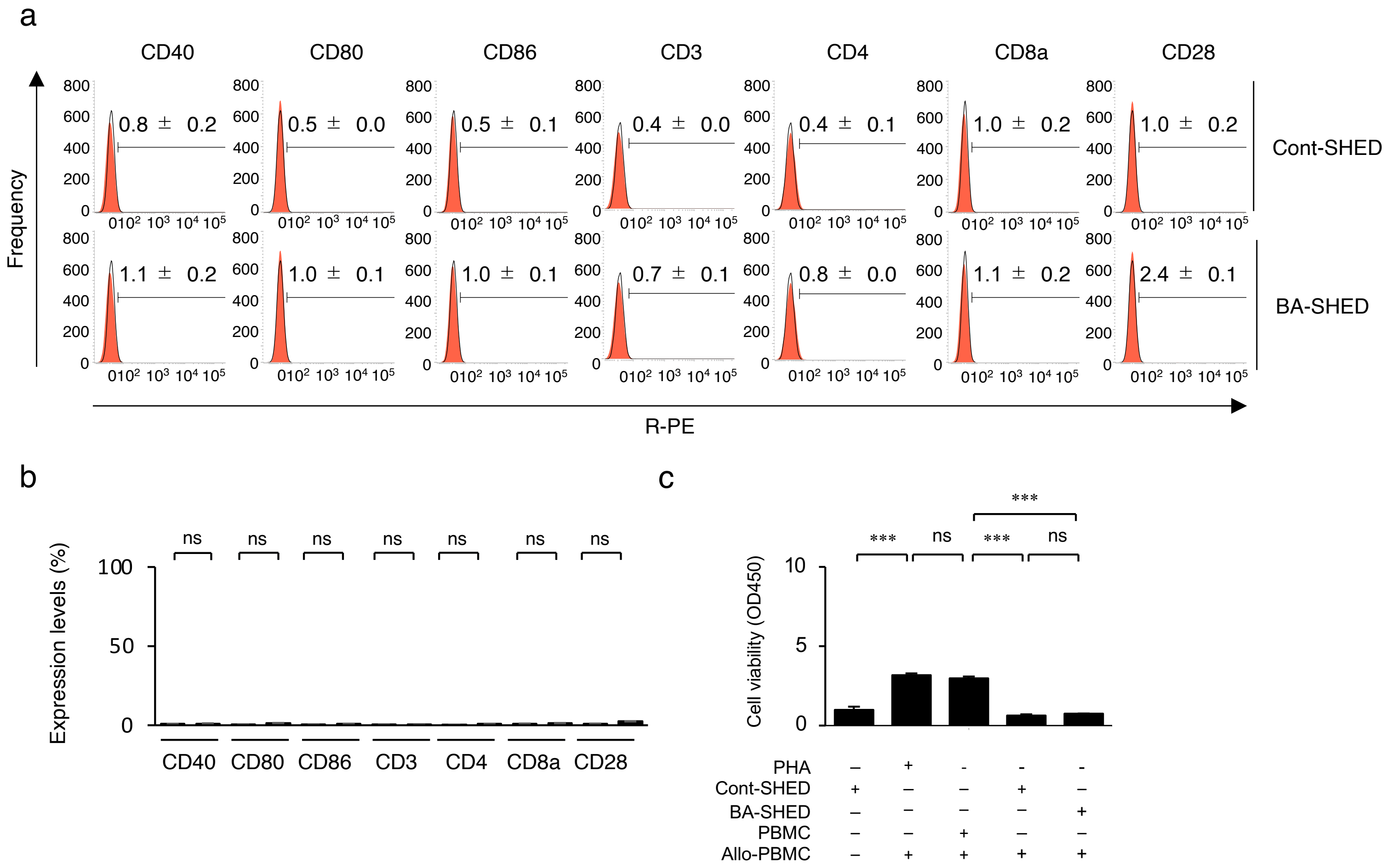

Supplementary Figure 2. Expression of antigenic surface antigens in BA-SHED and Cont-SHED.
(**a**) Representative histograms of antigenic surface antigens were analyzed by FCM analysis. The numbers indicate the means ± SEM of positive rate of target markers. Areas filled with red; target antibody-stained histograms; solid lines; isotype-matched control-stained histograms. (**b**) The graphs show the expression levels (%) of MSC markers in Cont-SHED and BA-SHED by FCM analysis. n = 3 for all groups. ns, no significance. The graph bars represent the means ± SEM. (**c**) Immunogenicity of Cont-SHED and BA-SHED was analyzed by mixed lymphocyte reaction assay. SHED and PBMCs were gamma-ray irradiated and cocultured with allogenic PBMCs (Allo-PBMCs) under the stimulation with or without phytohemagglutinin (PHA). Graph showing the cell viability of floating PBMNCs 3 days after coculture. n = 3 per group. Graph bars show the means ± SEM. *** $P < 0.005$. NS: no significance.

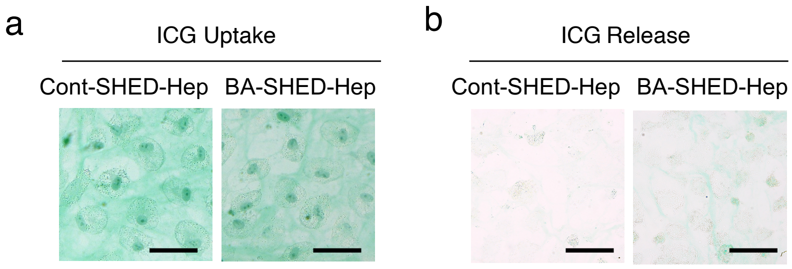

Supplementary Figure 3. Endocytotic and exocytotic capacity of hepatocyte like cells converted from BA-SHED (BA-SHED-Heps) and Cont-SHED (Cont-SHED-Heps). Representative images show indocyanine green (ICG) uptake (**a**) and release (**b**). Scale bars, 30 μm.

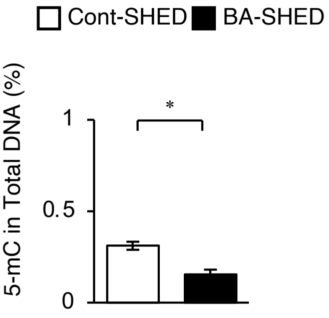

Supplementary Figure 4. Global methylation analysis of BA-SHED and Cont-SHED. The graphs show the 5-methylcytosine (5-mC) levels (%) in Cont-SHED and BA-SHED by enzyme-linked immunosorbent assay. n = 3 for all groups. $*P < 0.05$. The graph bars represent the means ± SEM.
